# PD‐1/PD‐L1 blockade rescue exhausted CD8+ T cells in gastrointestinal stromal tumours via the PI3K/Akt/mTOR signalling pathway

**DOI:** 10.1111/cpr.12571

**Published:** 2019-02-03

**Authors:** Rui Zhao, Yinghan Song, Yong Wang, Yuqian Huang, Zhigui Li, Yaping Cui, Mengshi Yi, Lin Xia, Wen Zhuang, Xiaoting Wu, Yong Zhou

**Affiliations:** ^1^ Department of Gastrointestinal Surgery, West China Hospital Sichuan University Chengdu China; ^2^ Department of Day Surgery Center, West China Hospital Sichuan University Chengdu China

**Keywords:** exhausted CD8+ T cell, gastrointestinal stromal tumour, PD‐1/PD‐L1, PI3K/Akt/mTOR

## Abstract

**Objectives:**

Although targeted therapy has revolutionized the treatment of gastrointestinal stromal tumours (GIST), it is almost never curative in GIST, and resistance commonly develops. One potential strategy is to combine targeted therapy with immunotherapy.

**Materials and methods:**

We first studied Programmed cell death 1 ligand 1 (PD‐L1) expression and tumour‐infiltrating T cells (TILs) in GIST. IFN‐γ was used to induce the upregulation of PD‐L1 expression in GIST‐882 cells, a well‐known GIST cell line. CD8+ T‐cell apoptosis was determined by flow cytometry. The PI3K/Akt/mTOR levels in CD8+ T cells were examined by Western blotting.

**Results:**

PD‐L1 expression was an independent factor of poor prognosis in GIST and resulted in exhausted T cells in the TILs population or the blood. Then, we found that PD‐L1 blockade alone could not increase tumour cell apoptosis in GIST. The apoptosis rate of CD8+ T cells was higher when T cells were cultured with PD‐L1+ GIST‐882 cells (GIST‐882 cells with high PD‐L1 expression) than when T cells were cultured with control GIST‐882 cells. However, when the PD‐L1 blockade was used, the apoptosis rates of the CD8+ T cells in the two groups became similar. Then, Western blotting showed the PI3K/Akt/mTOR levels of the CD8+ T cells rescued by the PD‐1/PD‐L1 blockade were higher than those of the CD8+ T cells not treated with the PD‐1/PD‐L1 blockade.

**Conclusions:**

PD‐L1 expression was an independent poor prognosis factor in GIST. PD‐1/PD‐L1 blockade rescued exhausted CD8+ T cells in GIST via the PI3K/Akt/mTOR signalling pathway. In GIST, PD‐1/PD‐L1 not only function as predictive biomarkers but also improve current therapies as treatment targets.

## 1 INTRODUCTION

Gastrointestinal stromal tumour (GIST) is the most common gastrointestinal soft tissue malignancy.^1^ Approximately 85% of GISTs contain an activating mutation in the *KIT* proto‐oncogene, whereas 5%‐10% have a mutation in the gene encoding *PDGFRA*.^1^, ^2^ Targeted molecular therapy has revolutionized the treatment of GIST and significantly improved the prognosis of GIST patients. In 2001, imatinib was first shown to treat metastatic gastrointestinal stromal tumours effectively, and it has improved the median overall survival of GIST patients from 9 months to over 5 years.^3^, ^4^, ^5^, ^6^, ^7^ However, imatinib is almost never curative in GIST, and resistance commonly develops at a median time of 18 months, mostly due to a secondary *KIT* or *PDGFRA* mutation.^6^, ^8^ Even though sunitinib and other new targeted drugs can sometimes be effective in recurrent GIST, clinical progression and drug resistance, such as insensitivity to sunitinib, subsequently evolve within 1 year.^9^, ^10^ Another potential strategy to increase the efficacy of imatinib is to combine imatinib with immunotherapy.

Many studies have confirmed that T cells, especially CD8+ T cells, a crucial component of the cellular immune response, are critical for the anti‐tumour effects of imatinib in GIST. T cells not only control a variety of bacterial and viral infections but also represent a major arm of the cell‐mediated anti‐tumour immune response.^11^ CD8+ T cells have been shown to play an important role in host defence and exhibit cytotoxicity against malignancies.^12^, ^13^ However, in cancer, CD8+ T cells upregulate the expression of inhibitory receptors, resulting in dysfunction and apoptosis in CD8+ T cells, which are then described as exhausted CD8+ T cells.^15^, ^16^, ^17^, ^18^ This process of exhaustion results in insufficient numbers of CD8+ T cells capable of killing tumour cells and leads to rapid tumour progression, including proliferation, invasion and metastasis.^19^ Programmed cell death protein 1 (PD‐1) has been shown to be expressed on exhausted T cells and to be a major mechanism of immune escape that malignancies take advantage of to evade destruction.^20^, ^21^ PD‐1 is a 288 amino acid protein that is expressed in activated mature T cells to regulate the balance between activating and inhibitory signals.^22^ Programmed cell death 1 ligand 1 (PD‐L1), the main ligand for Programmed cell death 1 ligand 1 (PD‐L1), is expressed on tumours and can lead to impaired T‐cell proliferation and effector functions, leading to apoptosis of tumour‐specific T cells.^22^, ^23^ In multiple solid malignancies, PD‐L1 is typically expressed on the surface of the tumour cells and appears to be upregulated, which helps tumour cells evade the cytotoxicity of T cells.^24^, ^25^ Thus, PD‐1/PD‐L1‐targeted therapies can enhance T‐cell responses and play a critical role in rescuing exhausted T cells by regulating costimulatory molecules.^26^, ^27^

A better understanding of the mechanisms of T‐cell exhaustion can provide novel therapeutic targets for the treatment of different tumours. Here, we have known that the PD‐1/PD‐L1 axis is a critical pathway leading to T‐cell exhaustion, with the expression of PD‐1 on CD8+ T cells correlating with a severely exhausted T‐cell response.^28^ However, the understanding of PD‐1/PD‐L1 therapies is still limited in GIST.^29^, ^30^ Overall, CD8+ T‐cell exhaustion mechanisms regulated by PD‐1/PD‐L1 in GIST remain largely undefined. In our study, we analysed the expression of PD‐L1 associated with tumour‐infiltrating T cells (TILs) and tumour biological characteristics in GIST. The frequency and functional characteristics of exhausted CD8+ T cells, which were identified based on their PD‐1 expression, were evaluated. To determine the effects of the PD‐1/PD‐L1 axis on CD8+ T cells in GIST, the correlation of exhausted CD8+ T cells with the expression of PD‐L1 was also addressed. Furthermore, we tested the combination of imatinib with PD‐1/PD‐L1 blockade on GIST cells and CD8+ T cells in vitro.

## 2 MATERIALS AND METHODS

### 2.1 Patient samples

Fresh‐frozen tumour tissue samples, normal gastric tissue samples, adjacent tumour tissue samples and matched peripheral blood samples were obtained from 238 GIST patients who underwent surgeries in West China Hospital, Sichuan University, and consented to the protocol approved by the Institutional Review Board.

### 2.2 Quantitative real‐time RT‐PCR was used to detect the expression of PD‐L1 mRNA

The RNA samples were quantified using an ultraviolet spectrophotometer (Beckman DU‐640; Beckman Instruments, Brea, CA, USA), and the RNA that met the RT reaction requirement of 1.8 < OD260/OD280 < 2 was used. Equal amounts of RNA were reverse transcribed using a High‐Capacity cDNA Reverse Transcription Kit (Applied Biosystems, Waltham, MA, USA). Through a search of GeneBank, we obtained the gene sequences of PDL1 (B7‐H1) and β‐actin. According to the principle of PCR primer design, the primers for PDL1 were designed with Primer 5.0 and had the sequences 5′‐GCCGAAGTCATCTGGACAAG‐3′ and 5′‐TCTCAGTGTGCTGGTCACAT‐3′, which were synthetized by Qing Ke Biological Engineering Company, Chengdu. PCR was performed with TaqMan gene expression master mix (Applied Biosystems) using 2 μL of cDNA in a 20 μL final reaction volume. The amplification cycles were performed by an ABI 7900HT real‐time PCR instrument (ABI Company, Waltham, MA, USA). The expression level of the housekeeping gene β‐actin (probe sequences: 5′‐CACCATCTTCCAGGAGCGAG‐3′ and 5′‐CCTTCTCCATGGTGGTGAAGAC‐3′) was used as an internal control to evaluate the integrity of each sample. The standard curve was drawn, and the quantitative fluorescence results were analysed by Bio‐Rad iq5 software.

### 2.3 Immunofluorescent staining analysis for the quantification of TILs

Immunohistochemistry (IHC) staining for the PD‐L1, CD4+ and CD8+ proteins was performed using the streptavidin‐peroxidase method (SP method). The paraffin‐fixed slides were dewaxed in xylene and rehydrated with 95% alcohol. PBS containing 10% normal goat non‐immune serum was used to block the sections for 1 hour at room temperature. Then, the sections were incubated with the primary antibodies, including a rabbit anti‐PD‐L1 polyclonal antibody (1:200, ab153991; Abcam, Cambridge, UK), rabbit anti‐human CD4 antibody (1:50) and mouse anti‐human CD8 antibody (1:50), at 25°C for 2 hours. After that, they were treated with 0.2% Triton X‐100 and incubated at 25°C for 1 hour. Then, they were incubated with a secondary antibody (Alexa Fluor 488‐labelled goat anti‐rabbit IgG, Alexa Fluor 647‐labelled goat anti‐mouse IgG, or Cy3‐labelled goat anti‐rat IgG) at 25°C for 1 hour. The slides were counterstained with haematoxylin and mounted under coverslips. The staining was quantified by manual counting and imagej software.

### 2.4 Cell lines

The GIST‐882 cell line (KIT exon 13 mutant; provided by Jonathan Fletcher) was maintained at 37°C in 5% CO_2_ in supplemented DMEM. The cells were incubated with recombinant human IFN‐γ (100 ng/mL; R&D Systems, Minneapolis, MN, USA) and imatinib (100 nmol/L; Novartis Basel, Switzerland).

### 2.5 Flow cytometry and cytokine detection

Human‐specific antibodies were purchased from Thermo Fisher Scientific (CD8) (Waltham, MA, USA) and Cell Signaling Technology (CD117) (Danvers, MA, USA). A viability dye was typically used to exclude dead cells. For flow cytometry, the GIST‐882 cell lines or CD8+ T cells were harvested, washed and then labelled with anti‐human CD117 or anti‐human CD8 for 30 minutes at 4°C in the dark. Then, all samples were measured by flow cytometry (Beckman Coulter). A 488 nm excitation wavelength was selected, FITC fluorescence was detected by a 515 nm wavelength filter, and PI was detected by a 560 nm wavelength filter. The results were analysed with flowjo software (version 7.6) (Ashland, OR, USA).

### 2.6 Western blot and cytokine array

Cells were harvested and suspended in RIPA lysis buffer containing 1 mmol/L phenylmethylsulfonyl fluoride (PMSF). Antibodies for p‐AKT (PS473), p‐PI3Kp85 (PY607), p‐s6 (Ser240/244)) and β‐actin were purchased from Cell Signaling Technology. Total protein was isolated from frozen tumour tissue samples and tested for cytokine/chemokine expression using a Proteome Profiler Array (R&D Systems). Densitometry was conducted on blots using imagej. Signal quantitation was calculated using Quantity One software (Bio‐Rad Laboratories, Hercules, CA, USA), and signals were normalized to the β‐actin signal.

### 2.7 Ethics statement

The study protocol was approved by the ethics committee of West China Hospital, Sichuan University. Written informed consent was obtained from the patients before beginning the study. Patient records/information were anonymized and deidentified prior to analysis, and the methods were adopted in accordance with the approved guidelines.

### 2.8 Statistical analyses

All statistical analyses were performed using spss version 20.0 (for Window; IBM, AMONG, NY, USA). We used the chi‐square test to compare categorical data and a *t* test or ANOVA to compare continuous data. The experimental data are expressed as the mean ± standard deviation (SD). The survival data were compared using the Kaplan‐Meier method and the log‐rank test to detect differences in the survival curves of the various groups. All *P* values were two‐tailed, and *P* values <0.05 were considered significant.

## 3 RESULTS

### 3.1 PD‐L1 expression in human GIST and tumour biological characteristics

**Figure 1:**
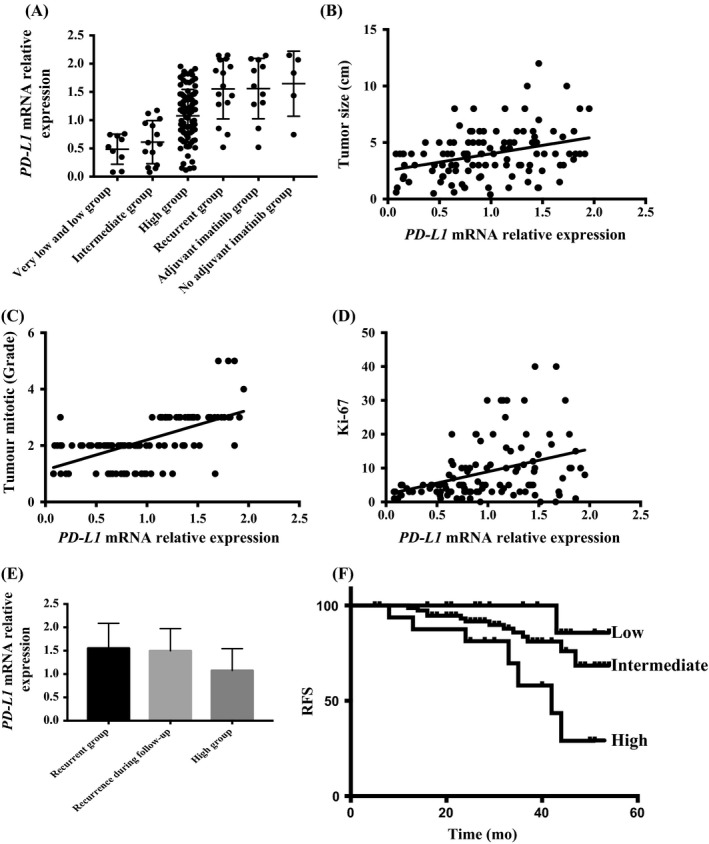
PD‐L1 expression in human GIST and tumour biological characteristics and prognosis. GIST, gastrointestinal stromal tumours

To assess the expression levels of PD‐L1 associated with tumour biological characteristics in human GIST, we evaluated 127 human specimens from patients who underwent surgeries for GIST in our hospital between January 2013 and December 2015. Of the 127 studied patients, 89 patients (70%) were classified as high risk, and 14 patients (11%) were intermediate risk. Only nine patients (7%) were diagnosed as low/very low risk. There were 15 patients (12%) who had recurrent GIST. Among them, 11 patients had received adjuvant imatinib over 1 year after the first surgery, and four patients did not undergo adjuvant imatinib. To identify PD‐L1 expression that was relevant in GIST, we performed quantitative real‐time PCR (qPCR) on freshly isolated tumour samples, which was a more accurate means of measuring the expression of PD‐L1 within the tumours than immunohistochemistry (Figure 1A).^31^, ^32^ The expression levels of PD‐L1 were variable and closely related to the modified NIH risk classification (Figure 1A). PD‐L1 expression was significantly lower in very low‐/low‐risk or intermediate‐risk GIST than in high‐risk GIST. PD‐L1 was expressed at the highest levels in recurrent GIST. Interestingly, when comparing PD‐L1 expression in recurrent GISTs from patients treated with adjuvant imatinib and from those without, there was no significant difference, suggesting that adjuvant imatinib did not impact the regulation of PD‐L1, which matched the results of previous research.^29^ PD‐L1 expression was positively correlated with tumour size, tumour mitotic rate and Ki‐67 expression in 112 samples, but not in the 15 recurrent GIST samples (Figure 1B‐D). However, interestingly, the PD‐L1 expression in tumours with different mutations, rupture statuses or sites was similar (data not shown), which was similar to the results of another study.^33^ While following 112 GIST patients for nearly 1‐5 years, tumour recurrence was observed in 21 patients, including 12 patients not treated with adjuvant imatinib and nine patients treated with adjuvant imatinib. For the follow‐up, these patients were divided into three groups according to their PD‐L1 expression: the low group (*PD‐L1 *mRNA relative expression <0.8); intermediate group (expression between 0.8 and 1.6); and high group (expression >1.6). In all patients with GIST, patients with high PD‐L1 expression showed a higher rate of relapse than those with low PD‐L1 expression, and the PD‐L1 expression in the 21 recurrent patients from the follow‐up study was similar to that of the 15 patients who relapsed before surgery and higher than that of the high‐risk GIST patients with low PD‐L1 expression, which showed that PD‐L1 expression affected clinical outcome in high‐risk GIST patients (Figure 1E). The rate of 5‐year relapse‐free survival (RFS) for patients with low PD‐L1 expression was 94.44% compared to 56.25% for patients with high PD‐L1 expression, and this difference achieved statistical significance (*χ* ^2^ = 7.28, *P* = 0.007, Figure 1F). The 5‐year RFS in the intermediate PD‐L1 expression group was also higher than that of the high PD‐L1 expression group (83.33% vs 56.25%, respectively, *χ* ^2^ = 5.83, *P* = 0.016, Figure 1F). However, the difference between the low PD‐L1 expression and intermediate PD‐L1 expression groups was non‐significant (*χ* ^2^ = 1.45, *P* = 0.23, Figure 1F). In a univariate analysis, PD‐L1 expression, tumour size, tumour mitotic index and risk classification were associated with the risk of relapse, whereas patient age, anatomical site and tumour mutations were not. In a multivariate analysis, PD‐L1 expression remained significant (*P* = 0.002), similar to the results of a previous study.^33^ The results illustrated that PD‐L1 expression was an independent poor prognosis factor in GIST.

### 3.2 PD‐L1 expression on tumour‐infiltrating/blood T cells in GIST

**Figure 2:**
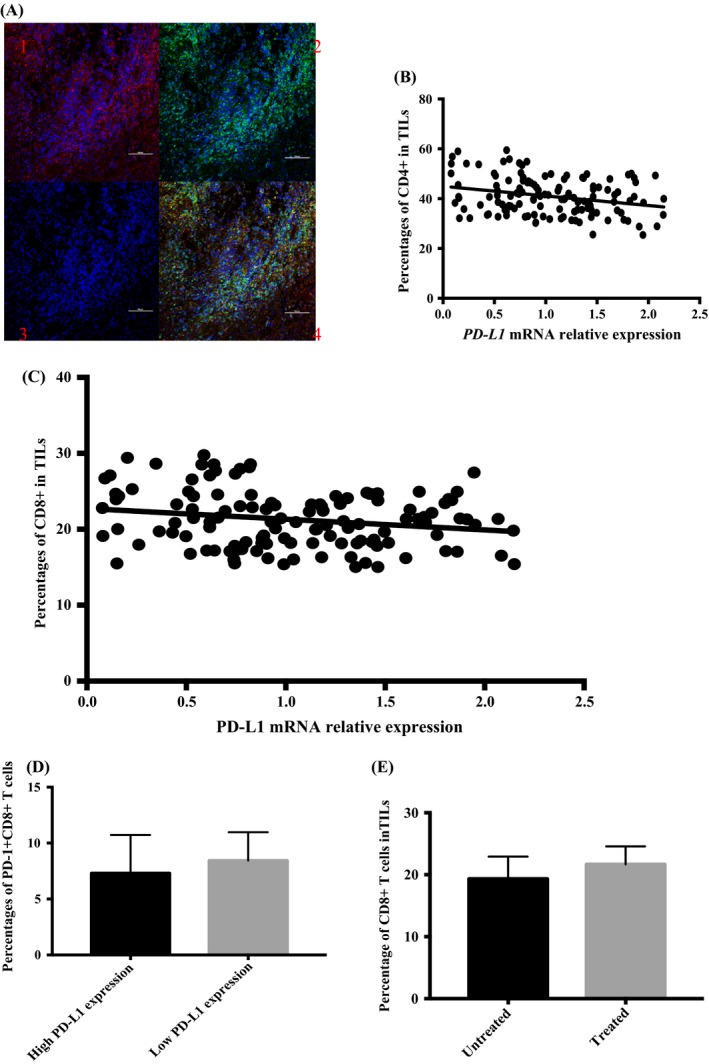
PD‐L1 expression and tumour‐infiltrating/blood T cells of human GIST. GIST, gastrointestinal stromal tumours

To investigate the response of tumour‐infiltrating/blood T cells to PD‐L1 on the surface of GIST tumours, we performed IHC and flow cytometry to identify tumour‐infiltrating/blood T cells from 127 GIST samples (Figure 2A). The median PD‐L1 expression was used as a cut‐off value. The percentages of CD4+ and CD8+ T cells were strongly and inversely correlated with PD‐L1 expression in human GIST (*F* = 9.90, *P* = 0.0021, *F* = 5.25, *P* = 0.024, Figure 2B,C).However, we found that the percentages of PD‐1+CD8+ T cells in the high PD‐L1 expression GISTs were lower than those in the low PD‐L1 expression GISTs, which suggested that sustained high expression of PD‐L1 characterized environments with exhausted CD8+ T cells (*P* = 0.036, Figure 2D).^34^ Interestingly, the percentage of CD8+ T cells in GISTs treated with imatinib was slightly higher than that in untreated GISTs, illustrating that imatinib might induce a dramatic increase in the number of CD8+ T cells (*P* = 0.041, Figure 2E). Moreover, in the blood, the percentages of CD4+ or CD8+ T cells were also inversely associated with the PD‐L1 expression level, which is the same as what was observed in the tumours. These results suggest that exhausted T cells exist in GIST and are related to the PD‐L1 expression of the tumour.

### 3.3 PD‐L1 blockade in GIST‐882 cells and IFN‐γ‐induced upregulation of PD‐L1 expression on GIST‐882 cells

**Figure 3:**
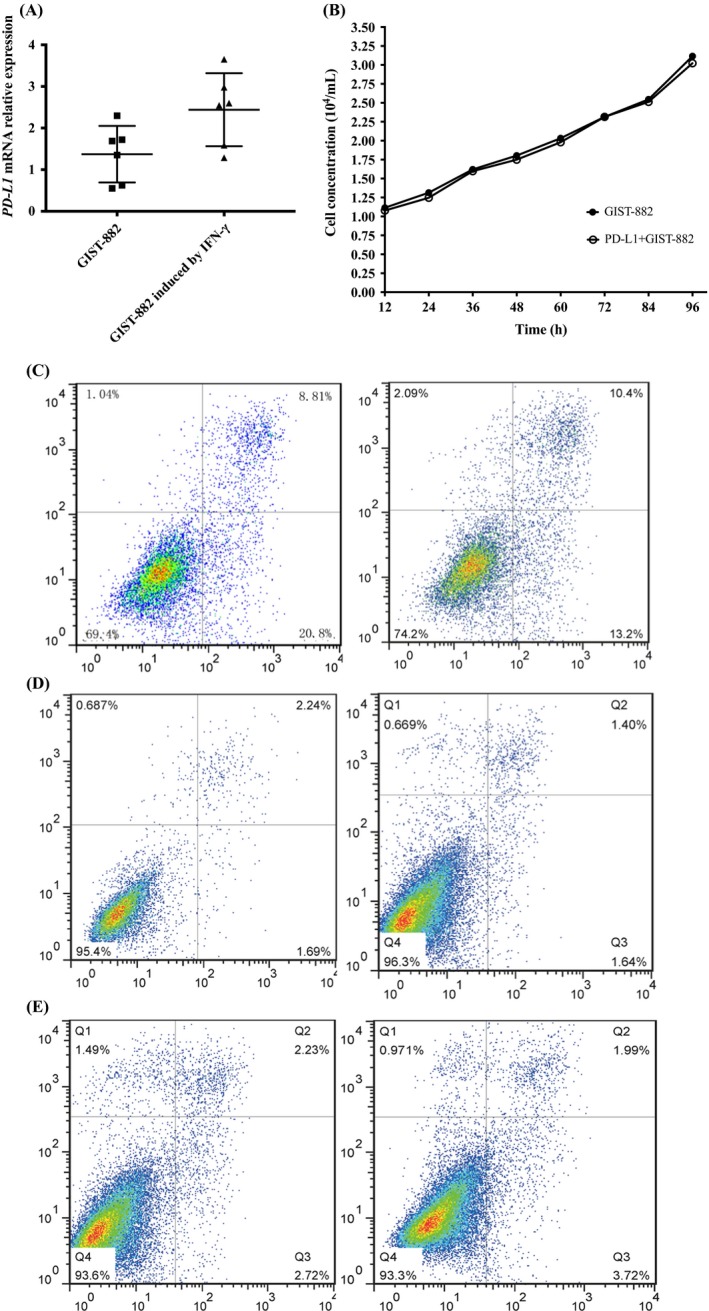
PD‐L1 blockade for GIST‐882 and IFN‐γ‐induced upregulation of PD‐L1 on GIST‐882. GIST, gastrointestinal stromal tumours

Previous studies have reported that IFN‐γ can induce PD‐L1 expression in GIST.^35^ Thus, to determine how PD‐L1 regulates CD8+ T cells, we administered IFN‐γ to the GIST‐882 cell line to induce the upregulation of PD‐L1 expression. IFN‐γ could upregulate PD‐L1 expression over time as measured by qPCR (Figure 3A). Next, to determine whether PD‐L1 expression would affect the proliferative ability of two cell lines, we assessed proliferation in GIST‐882 and PD‐L1+GIST‐882 (GIST‐882 cells with high PD‐L1 expression) cells by a CCK‐8 assay (Figure 3B). As expected, similar proliferation was observed with GIST‐882 and PD‐L1+GIST‐882 cells. To further confirm the effect of PD‐L1 blockade on the proliferative ability of tumours, we added imatinib to GIST‐882 and PD‐L1+GIST882 cells, and the apoptosis rates of the two cell lines were not significantly different by flow cytometry, suggesting that the sensitivities of the two cell lines to imatinib were similar (Figure 3C). To determine whether M1H1 can be used alone to treat GIST, we added M1H1 alone to a group of PD‐L1+GIST‐882 cells, and another group was cultured without M1H1. Comparing apoptosis in the two groups by flow cytometry, there was no obvious difference in the rates of apoptosis of the groups with or without PD‐L1 blockade (Figure 3D,E). The PD‐L1 blockade alone could not increase tumour cell apoptosis due to the high PD‐L1 expression in human GIST cells in vitro, and the same result was obtained in a clinical trial.^30^

### 3.4 PD‐L1 blockade for exhausted CD8+ T cells cultured with GIST‐882 cells in vitro

**Figure 4:**
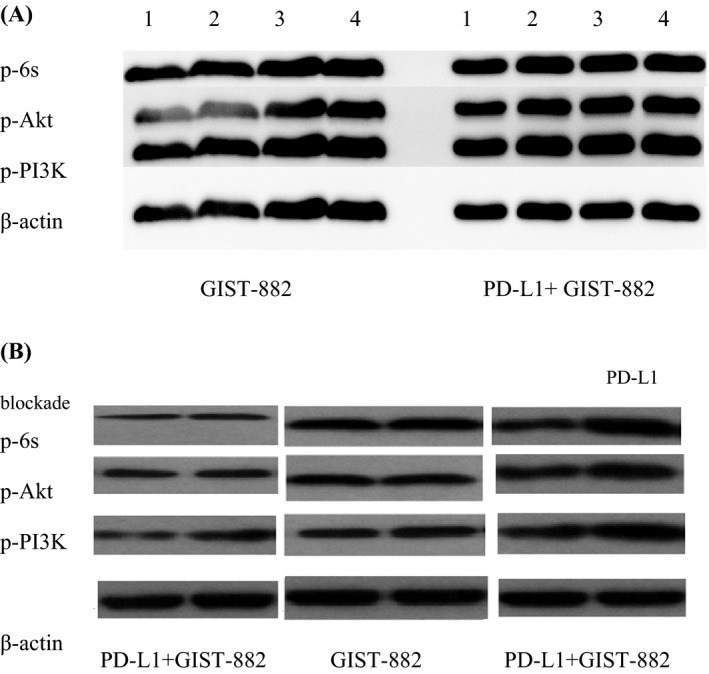
PD‐L1 blockade for tumour and exhausted CD8+ T cells in GIST‐882 or PD‐L1+GIST‐882 in vitro. GIST, gastrointestinal stromal tumours

To explore the effect of PD‐L1 blockade on CD8+ T cells cultured with GIST‐882 cells in vitro, GIST‐882 cells or PD‐L1+GIST‐882 cells and CD8+ T cells were cultured together at a ratio of 1:20 in vitro as different experimental groups. The four experimental groups were GIST‐882 and CD8+ T cells (Group A); PD‐L1+GIST‐882 and CD8+ T cells (Group B); GIST‐882 cells, CD8+ T cells and M1H1 (Group C); and PD‐L1+GIST‐882 cells, CD8+ T cells and M1H1 (Group D). Comparing the results of the groups, the rate of CD8+ T‐cell apoptosis in Group B was the highest among the rates in the four groups. Interestingly, the rates in Group A, Group C and Group D were similar, even if that in Group C was lower than that in Group A and Group D, but the difference was not significant. To determine whether CD8+ T cells could impact the effect of imatinib on GIST, we added imatinib to Group A and Group B to assess apoptosis in GIST tumour cells by flow cytometry. The apoptosis rate of Group A was higher than that of Group B, suggesting that CD8+ T cells contributed substantially to the anti‐tumour effect of imatinib (data not shown). To further identify the regulatory role of PD‐L1 blockade and CD8+ T cells in GIST, we also used a CCK‐8 assay to plot the cell growth curves of GIST cells in the different groups (data not shown). Comparing Group A and Group C, similar proliferation of the tumour cells was observed (data not shown), suggesting that PD‐L1 was ineffective in the context of low PD‐L1 expression on GIST‐882 cells. To further confirm the effect of the PD‐L1 blockade on the CD8+ T cells in the PD‐L1+GIST‐882 culture, we compared the results of Group B and Group D and found that the proliferation rate of the GIST cells in Group B was higher than that in Group D (data not shown). In the results, the difference between Group A and Group B suggested that high PD‐L1 expression could decrease the activity of the CD8+ T cells and increase the number of exhausted CD8+ T cells. By comparing Group C and Group D, we found that the PD‐L1 blockade was more effective in the high PD‐L1 expression group. Next, to directly determine the CD8+ T‐cell–intrinsic requirement for PI3K/Akt/mTOR signalling in PD‐L1‐mediated rescue, we examined p‐PI3K, p‐Akt and p‐6s expression in the CD8+ T cells and tumour cells of the four groups by Western blotting (Figure 4A,B). First, we performed the PD‐L1 blockade with CD8+ T cells alone or added together with GIST‐882 and PD‐L1+GIST‐882 cells. The results showed that the PD‐L1 blockade and/or presence of CD8+ T cells did not affect the protein expression of p‐PI3K, p‐Akt and p‐6s in the tumour cells (Figure 4A). Then, we tested the CD8+ T cells from the different groups. Western blotting confirmed that the p‐PI3K, p‐Akt and p‐6s proteins were expressed at higher levels in Group D than in Group B, but there was no significant difference between Group A and Group C, even though the levels in Group C were slightly higher (Figure 4B).

## 4 DISCUSSION

Tyrosine kinase inhibitors, including imatinib and sunitinib, are effective in GIST but often have only transient benefits as resistance commonly develops. Immunotherapy, particularly PD‐1/PD‐L1 blockade, has shown efficacy in a variety of cancers.^36^, ^37^, ^38^, ^39^, ^40^ However, not all patients have derived clinical benefits from PD‐1/PD‐L1 therapy, and the molecular mechanisms underlying the regulation of T cells in GIST are not elucidated. In tumour immunity, CD8+ T cells play a critical role in resisting cancers and are one of the main types of T cells regulated by PD‐1/PD‐L1. Thus, it is critical to determine the mechanism by which PD‐1/PD‐L1 regulates CD8+ T cells to improve the design of novel CD8+ T‐cell–based immunotherapies.

In this study, we found that the PD‐1/PD‐L1 axis contributed to immune evasion and predicted prognosis in GIST. Notably, the PD‐L1 expression on GIST tumour cells within different risk classifications was variable, and a distinct population of high‐risk and resistant GIST patients had very high PD‐L1 expression, suggesting that PD‐1/PD‐L1 blockade might particularly benefit a subset of patients who urgently need to receive post‐operative treatment. The analysis of the correlations between PD‐L1 mRNA expression and tumour features showed that the upregulation of PD‐L1 expression was associated with poor prognosis features (high risk, a high tumour mitotic index and a high proliferation rate), which is in agreement with the results of previous publications.^29^, ^33^ This indicates that the malignancy of tumours is related to the expression of PD‐1, but the causal relationship between them is not clear. Furthermore, PD‐L1 expression was also higher in recurrent GIST than in high‐risk GIST, and the median RFS of patients with low PD‐L1 expression was longer than that of patients with high PD‐L1 expression, suggesting PD‐L1 expression has an unfavourable prognostic value. This was confirmed in the univariate and multivariate analyses, which identified not only the four factors included in the modified NIH classification but also the PD‐L1 expression level as independent prognostic factors. The PD‐L1 expression results of patients who underwent adjuvant imatinib were similar to those of patients who did not receive treatment after surgery. To our knowledge, this is the first report analysing PD‐L1 expression in GIST. Our results support the notion that imatinib exerts beneficial off­target effects on the immune system and enhances anti‐tumour T‐cell responses in GIST and chronic myeloid leukaemia.^41^ In future studies, we will examine the effect of imatinib on different subtypes of lymphocytes in biopsies to explore a new approach for GIST treatment.

Next, by comparing the PD‐L1 expression and TIL numbers, we discovered that the PD‐L1 expression in tumours was strongly interrelated with the number of TILs, matching reports from other studies, suggesting that PD‐L1 expression can limit the immune response to GIST in the tumour microenvironment.^33^, ^42^, ^43^ A number of studies support the notion that TILs in the tumour microenvironment are associated with prognosis in a range of cancers and exhibit reactivity against tumours in the tumour microenvironment.^44^, ^45^, ^46^, ^47^ The upregulation of PD‐L1 expression in tumours could initiate the mechanism of “adaptive immune resistance,” which could lead to the selective suppression of tumour‐specific T cells to protect the tumour cells from attacks by the immune system.^20^ PD‐L1 expression can affect the prognosis of patients with GIST by inhibiting TILs, especially CD8+ T cells, to avoid immune cell attacks. We observed that the percentages of PD‐1+CD8+ T cells in high PD‐L1 expression GISTs were lower than those in low PD‐L1 expression GISTs. This result occurs because the bonding between PD‐1+CD8+ T cells and PD‐L1+ tumour cells rapidly destroys the activity of PD‐1+CD8+ T cells, producing TILs described as exhausted CD8+ T cells. In addition, the CD4+ or CD8+ T‐cell numbers were decreased in GIST patients with high PD‐L1 expression in their tumours, suggesting that the exhausted T cells occurred not only in the tumour microenvironment but also in the whole body. This observation is explained by the latest study, which reports that cancer cells can send out “drones,” extracellular vesicles mostly in the form of exosomes that carry PD‐L1 on their surface, to battle the immune system from afar.^48^ That study unveils a mechanism by which tumour cells systemically suppress the immune system, which might also occur in GIST.

Multiple results have been shown for PD‐L1 blockade in GIST cells. First, IFN‐γ can induce PD‐L1 expression, likely through STAT1, which has been reported in many studies.^49^, ^50^ However, the proliferative abilities of GIST‐882 and PD‐L1+GIST‐882 cells were similar, suggesting that PD‐L1 expression may be induced by many cytokines and immune cells and can partly reflect the immune activity in patients. Thus, PD‐L1 expression was identified as a predictive biomarker. Furthermore, there was no obvious difference in the apoptosis of PD‐L1+GIST‐882 cells with or without the PD‐L1 blockade. This can explain why of all patients who receive PD‐1/PD‐L1 blockade, only a subset of patients benefit from the therapy, and the majority of patients are primarily resistant to PD‐1/PD‐L1 blockade. The PD‐1/PD‐L1 axis cannot regulate tumour cell death but is a key inhibitory checkpoint that alters the function of T cells after antigen‐mediated stimulation. Thus, when CD8+ T cells are lacking inside tumour lesions, patients do not respond to PD‐1/PD‐L1 blockade therapy.^51^ If CD8+ T cells inhibited by PD‐1/PD‐L1 are present in inadequate numbers within the tumour microenvironment, then PD‐1/PD‐L1 blockade therapy would be unlikely to work.^49^

The CD8+ T‐cell apoptosis rate was higher in PD‐L1+GIST‐882 cultures than in GIST‐882 cultures, and when the PD‐L1 blockade was added, the apoptosis rates of the CD8+ T cells in the two groups were similar. This illustrates that the PD‐1/PD‐L1 axis plays a major role in CD8+ T‐cell exhaustion in GIST. T‐cell exhaustion is a state of T‐cell dysfunction characterized by diminished cytokine production, impaired killing and hypoproliferation. In GIST, CD8+ T cells are critical and required for the maximal anti‐tumour effects of imatinib.^43^ Imatinib not only works via direct effects on tumour cells but also relies indirectly on the immune system.

PD‐1/PD‐L1 blockade can enhance the anti‐tumour effect of imatinib by rescuing exhausted CD8+ T cells in GIST. It might be good news for patients with resistant or metastatic GIST that combined PD‐1/PD‐L1 blockade and imatinib could rapidly and efficaciously control the progression of disease. By measuring the levels of p‐PI3K, p‐Akt and p‐6s, we confirmed that the PD‐1/PD‐L1 blockade could rescue exhausted CD8+ T cells via the PI3K/Akt/mTOR signalling pathway in GIST cells with high PD‐L1 expression. The cell proliferation–related signal transduction pathway PI3K/Akt/mTOR is involved in the regulation of a variety of cell proliferation and apoptosis functions; thus, aberrantly elevated mTOR activity is frequently observed in human malignancies.^52^ However, mTOR signalling also plays a crucial role in both the innate and adaptive immune systems. Activated by the phosphorylation of PI3K/Akt/mTOR, mTOR can affect the cell cycle and regulate apoptosis by mediating the important downstream signal, which is of great significance in the exploration of the signalling pathway.^53^ Notably, AKT‐independent metabolic responses have been identified in CD8+ T cells.^54^, ^55^ Previous studies have shown that the activation of the PI3K/Akt/mTOR pathway can increase nutrient uptake and energy production in CD8+ T cells.^56^, ^57^ Increasing evidence suggests that mTOR plays a central role in regulating the biological outcomes of immune cell stimuli.^58^, ^59^, ^60^ In our study, we found that the PD‐1/PD‐L1 blockade could decrease apoptosis in the CD8+ T cells in vitro via the PI3K/Akt/mTOR signalling pathway, which was first reported in GIST. This mechanism in GIST could help us to discover more accurate therapies and new combination regimens to combat treatment side effects and resistant GIST, which constantly interfere with the treatment of GIST. Immunotherapy using PD‐1/PD‐L1 blockade is now widely used for the treatment of many malignant tumours, but only a subset of patients respond to the therapy.^39^, ^40^, ^61^ The mechanism by which PD‐1/PD‐L1 regulates CD8+ T cells found in our study may resolve the problem of non‐responding patients and allow the combined treatment to achieve effective clinical effects in GIST as soon as possible. However, our results are limited due to a lack of data from pre‐clinical mouse models. In the future, we will establish a mouse model to explore the PI3K/Akt/mTOR pathway in vivo.

Taken together, our study elucidated that PD‐1/PD‐L1 blockade rescued exhausted CD8+ T cells in GIST via the PI3K/Akt/mTOR signalling pathway. PD‐L1 expression was an independent poor prognosis factor in GIST. High PD‐L1 expression resulted in exhausted CD8+ T cells. PD‐L1 increased the efficacy of imatinib by recusing exhausted CD8+ T cells. In GIST, PD‐1/PD‐L1 not only acted as predictive biomarkers but also improved current therapies. The data provide greater insight into the mechanism involved in T‐cell exhaustion and the promotion of the effect of PD‐1/PD‐L1 blockade.

## CONFLICT OF INTEREST

All the authors declare that they have no relevant conflicts of interest to disclose.

## AUTHOR CONTRIBUTION

Prof. Yong Zhou, Yinghan Song and Rui Zhao conceived the study together. All authors contributed to the research and development process that resulted in the article. Rui Zhao, Yinghan Song, Yong Wang, Yuqian Huang, Zhigui Li, Mengshi Yi, Yaping Cui and Lin Xia performed the experiments. Rui Zhao and Yinghan Song wrote the manuscript under the guidance of Prof. Yong Zhou and Prof. Xiaoting Wu. All authors read the manuscript and approved the final manuscript.
